# Ductal Carcinoma In Situ With Microinvasion Arising in a Breast Hamartoma: A Case Report and Review of Guidelines of Hamartoma Management

**DOI:** 10.1155/cris/1681410

**Published:** 2026-10-27

**Authors:** Buket Ertansel, Geetha Joseph, Oniaza Syed, Anna Kirby, Marina Parton, Sarah Shuk-Kay Tang

**Affiliations:** ^1^ Department of Breast Surgery St. George’s Hospital NHS Foundation Trust Blackshaw Road SW17 0QT London UK nhs.uk; ^2^ Department of Histopathology Kingston Hospital Kingston and Richmond NHS Foundation Trust Kingston Upon Thames KT2 7QB, Galsworthy Rd London UK kingstonhospital.nhs.uk; ^3^ Department of Clinical Radiology Kingston Hospital Kingston and Richmond NHS Foundation Trust Galsworthy Rd, Kingston Upon Thames KT2 7QB London UK kingstonhospital.nhs.uk; ^4^ Royal Marsden Institute of Cancer Research London UK icr.ac.uk; ^5^ Kingston Hospital Kingston and Richmond NHS Foundation Trust Galsworthy Rd, Kingston Upon Thames KT2 7QB London UK kingstonhospital.nhs.uk

## Abstract

Breast hamartomas are rare, benign lesions composed of adipose, fibrous and glandular tissue. While typically non‐malignant and not linked to increased breast cancer risk, they are often underdiagnosed due to variable imaging and histological features. We report a rare case of ductal carcinoma in situ (DCIS) with microinvasion arising within a mammary hamartoma. Unexpectedly the patient went on to develop a triple‐negative invasive breast cancer 6 months following nipple‐sparing mastectomy and implant reconstruction. To date, only 16 cases of breast carcinoma within hamartomas have been reported, making this case an important addition to the limited literature. It highlights the need for careful imaging interpretation, pathological review and multidisciplinary management, particularly in lesions with glandular components or atypical features. With increasing use of breast imaging, identification of hamartomas is expected to rise, making awareness of their rare malignant potential essential. In the absence of prevalence data or management guidelines, this case underscores the value of centralised reporting and collaborative clinical decision‐making.

## 1. Introduction

Hamartoma of the breast is a local malformation made up of an abnormal mixture of cells and fatty, glandular and fibrous tissue. They tend to be well circumscribed masses that form due to disordered replications of normal tissue cells [1, 2]. These uncommon benign breast lesions were first described in the 1970 s [3] by Arrigoni et al. [2], their reported incidence in the literature is between 0.7% and 8% of all benign breast tumours in females [4, 5]. There are characteristic mammographic and sonographic appearances of some hamartomas; however, depending on their main tissue component (e.g.,fat or fibrous tissue), it can mimic other lesions such as lipomas or fibroadenomas. Similarly, microscopic diagnoses can often conclude alternative benign lesions or reveal normal breast tissue, and for these reasons, it is felt that hamartomas are under‐reported and underdiagnosed [6]. However, with the increased use of breast diagnostics, the number of hamartomas discovered is growing [7].

A few years after the work of Arrigoni et al. [2], Davies and Riddell [8] described a further subtype, the myoid (muscular) hamartoma, characterised by the presence of smooth muscle cells. This rare variant accounts for ~3.7% of breast hamartomas. Historically, breast hamartomas were regarded as developmental malformations rather than true neoplasms. However, molecular analysis and genetic studies demonstrating the high‐mobility group AT‐hook 2 (HMGA2) gene (an architectural transcription factor) rearrangements and corresponding protein overexpression provide strong evidence that the myoid subtype represents a genuine clonal neoplasm, challenging the traditional view of hamartomas as purely developmental lesions. This highlights the importance of genetic characterisation of such lesions and could determine whether it shares molecular features with other benign mesenchymal tumours [9, 10].

The literature has described many large case series, including over 150 case reports of hamartomas, their presentation, diagnostic reporting and their benign nature [7]. Hamartomas are not deemed to be an increased relative risk factor for breast cancer, and neither are they thought to usually undergo malignant transformation. However, there have been several reports of coexistence of carcinoma associated with breast hamartomas, which could suggest that malignant transformation could possibly occur, as there is a presence of glandular tissue in the lesion [11, 12] that may require closer inspection when these lesions are found. To date, there have only been 16 published cases of hamartomas with associated malignancy [13–15], giving us a small insight into the nature of how these cases have evolved and are treated.

As screening and detection of breast lesions continue to increase the incidence of these lesions may become more prevalent; we aim to provide further understanding of hamartomas associated with carcinoma with our case report. We share our multidisciplinary team’s (MDT) experience of a patient who was being monitored for a giant fibroadenoma and was subsequently found to have a ductal carcinoma in situ (DCIS) arising in a mammary hamartoma.

## 2. Case Report

Patient H is a 39‐year‐old fit and well female who presented with a left breast mass; she had previously been diagnosed with a 7 cm fibroadenoma on biopsy 15 years ago outside of the UK. Her breast surgeon had recommended excision of the lesion upon completion of her family and breastfeeding. She was monitored every 6 months up until 2 years ago, when she moved to the UK. Patient H presented again to a breast surgeon with a primary complaint of worsening premenstrual breast pain; she reported no other red flag breast symptoms. On examination, a large round lump immediately behind the left nipple areola complex was found with focal tenderness medial to the nipple, there was no evidence of skin or nipple changes; both axillae revealed no palpable lymph nodes and the contralateral right breast examination was normal.

Patient H has no significant past medical history and is on no regular medication. Her menstrual cycle is irregular, she does not use any hormonal contraception or therapy. Patient H has had two children aged 8 and 12 years old; they were both breast‐fed for a duration of 1 year. There was no familial history of breast cancer.

As part of the one‐stop assessment, the patient underwent a mammogram and ultrasound. The mammogram with further tomosynthesis revealed a large central hamartoma measuring up to 90 mm. Within this area, there was a well‐defined density measuring 20 mm and two clusters of pleomorphic microcalcification which were indeterminate; the largest cluster of the central outer breast measures 16 mm and the smaller cluster in the upper inner breast measures 9 mm (Figure 1).

**Figure 1:**
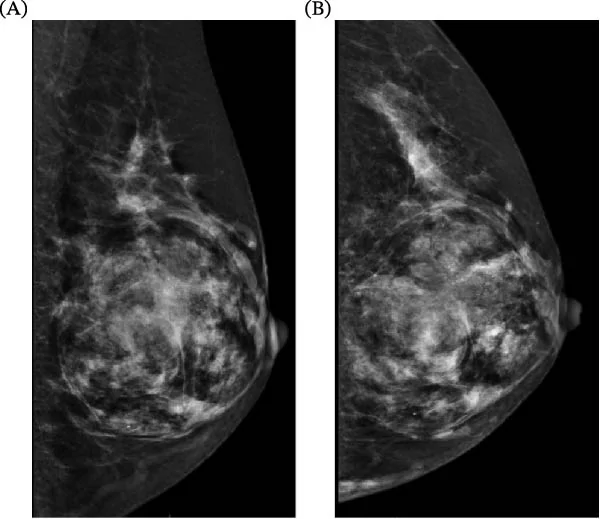
Mammogram left breast MLO view (A) and CC view (B). This shows a large centrally located known hamartoma measuring up to 90 mm; however, with associated pleomorphic calcification and an impression of distortion centrally within the mass. Tomosynthesis and magnification views performed which showed distortion within this mass and associated pleomorphic microcalcification. Mammogram and tomosynthesis graded as left M4/DBT4.

Satisfactory appearances were found in the right breast. The left breast was classified as Breast Imaging Reporting and Data System (BI RADS) 4 and therefore suspicious for cancer. Ultrasound of the left breast shows a large heterogeneous hypoechoic lesion in the central breast, with an approximate measurement of 70 mm. Heterogeneous changes are seen within 3 mm of the nipple base (Figure 2).

**Figure 2:**
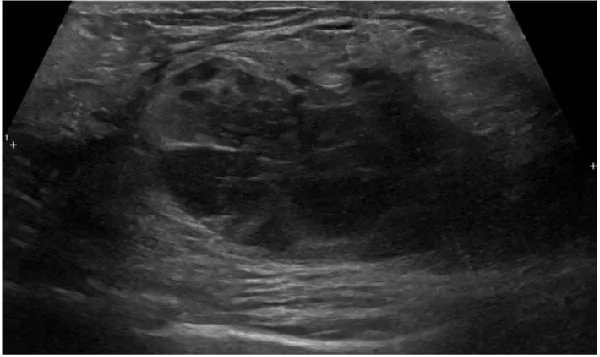
Ultrasound left breast. Ultrasound showed a 7.5 cm centrally located irregular hypoechoic mass with interval foci of calcification which corresponded to the areas of pleomorphic calcification on the mammogram. Ultrasound guided biopsy performed with calcification retrieved in the pathology specimen.

In the upper inner quadrant, there was an 18 mm × 13 mm hypoechoic lesion present; however, a linear hypoechoic tract is seen connecting this to the main mass centrally. Satisfactory appearances of the left axilla and of the right breast were concluded. Mammographic images were read and reported and then checked for concordance by two consultant breast radiologists as per local guidelines.

Core biopsy was undertaken of the suspicious lesion; it revealed a high‐grade solid type DCIS with a small focus of microinvasion, oestrogen receptor (ER) 0‐negative, progesterone receptor (PR) 0‐negative and they were unable to classify the human epidermal growth factor receptor (HER) 2 status due to the small size of the biopsy. Further tissue sampling was advised due to the large nature of the mass, as an invasive component may still be discovered, which could subsequently affect the need for sequencing and neoadjuvant treatments. Second site biopsy shows DCIS (no microinvasion). Immunohistochemistry staining was conducted and reported as per national guidance.

The pathology and imaging were discussed as part of the local breast MDT, and mastectomy was recommended. All options for mastectomy were discussed with the patient (flat, implant and autologous reconstruction); after the patient had time to review her options, she decided to undergo a unilateral nipple‐sparing mastectomy and direct to implant‐based and acellular dermal matrix (ADM) breast reconstruction and sentinel lymph node biopsy. No frozen sections were performed.

The final mastectomy post‐operative pathology was 120 mm × 120 mm × 50 mm in dimension and weighed 251 g. There was a circumscribed mass measuring 60 mm × 43 mm × 90 mm,;within this in the central breast, there is a hard lobulated, pale white, granular lesion measuring 50 mm × 40 mm × 25 mm (Figures 3, 4 and 5). Histopathological examination of all biopsies and specimens demonstrated no smooth muscle component; therefore, none of the lesions were classified as the myoid subtype. Informed consent was obtained for this case report.

**Figure 3:**
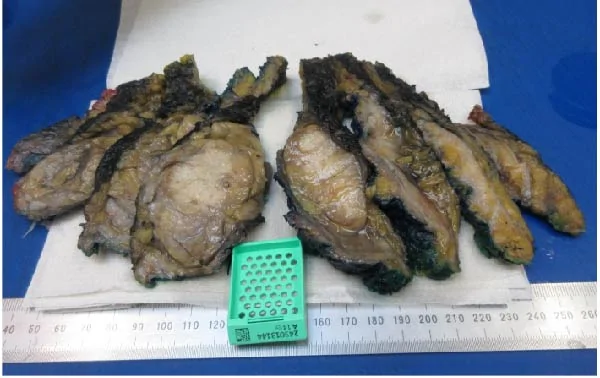
Histopathology of breast tissue. Mastectomy sample fixed in formaldehyde.

**Figure 4:**
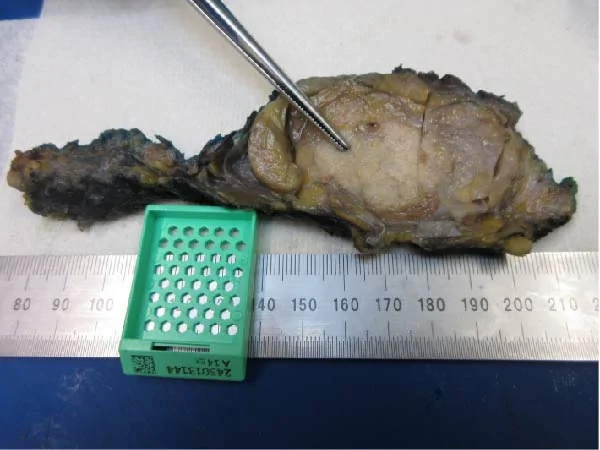
Histopathology of breast tissue. Area indicated by forceps is the DCIS.

**Figure 5:**
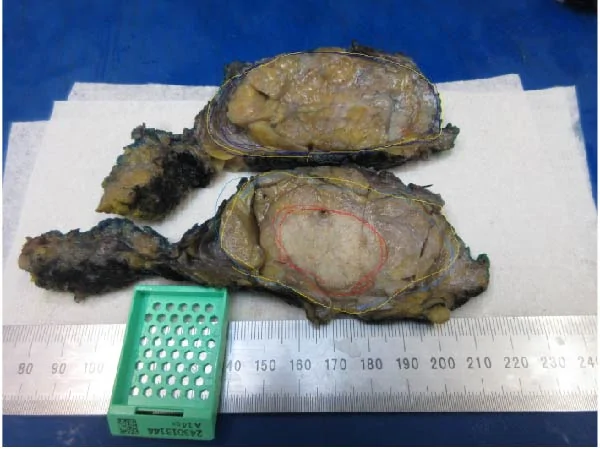
Histopathology of breast tissue. Area outlined in blue and yellow is the hamartoma, the red outline indicates the DCIS.

Sections of breast tissue from the 5 cm hard pale lesion described within the larger mass show confluent mass forming high‐grade solid DCIS with extensive comedo necrosis. There was no morphological evidence of microinvasion or invasive malignancy in the mastectomy specimen, and p40 and CK5 immunohistochemistry shows retained myoepithelial around all the proliferations, confirming the in situ nature of the lesion (Figures 6, 7 and 8). The lesion was closest to the deep margin and more than 10 mm away from all other margins, including the anterior skin margin. The tissue adjacent to the DCIS shows features consistent with the previously diagnosed hamartoma.

**Figure 6:**
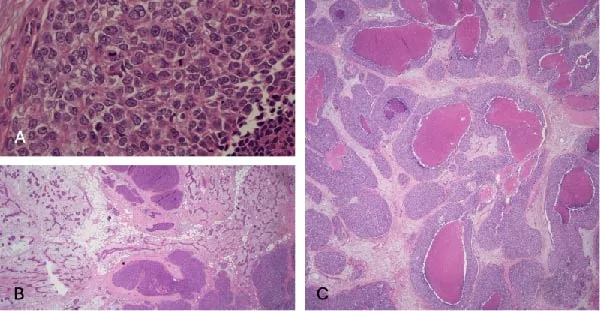
Microscopy images of breast specimen. (A) DCIS higher power highlighting mitoses, and comedo necrosis. (B) DCIS with hamartoma. (C) DCIS—larger field.

**Figure 7:**
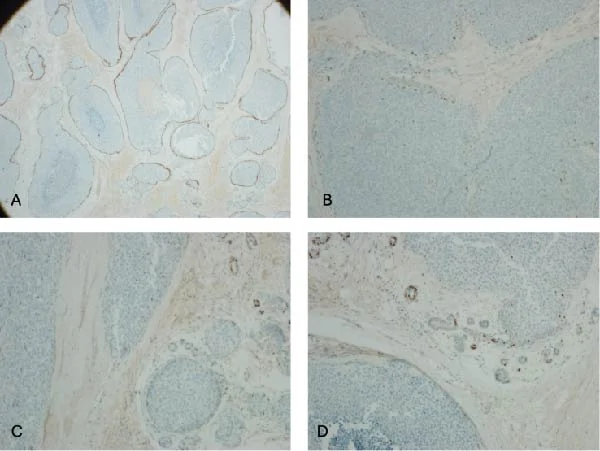
Further microscopic images. (A) DCIS with retained CK5 positive myoepithelium around the duct periphery. (B) DCIS with retained p40 positive myoepithelium around the duct periphery. (C) DCIS, PgR negative, epithelium of hamartoma is PgR positive. (D) DCIS is ER negative, benign epithelium of hamartoma shows ER positivity.

**Figure 8:**
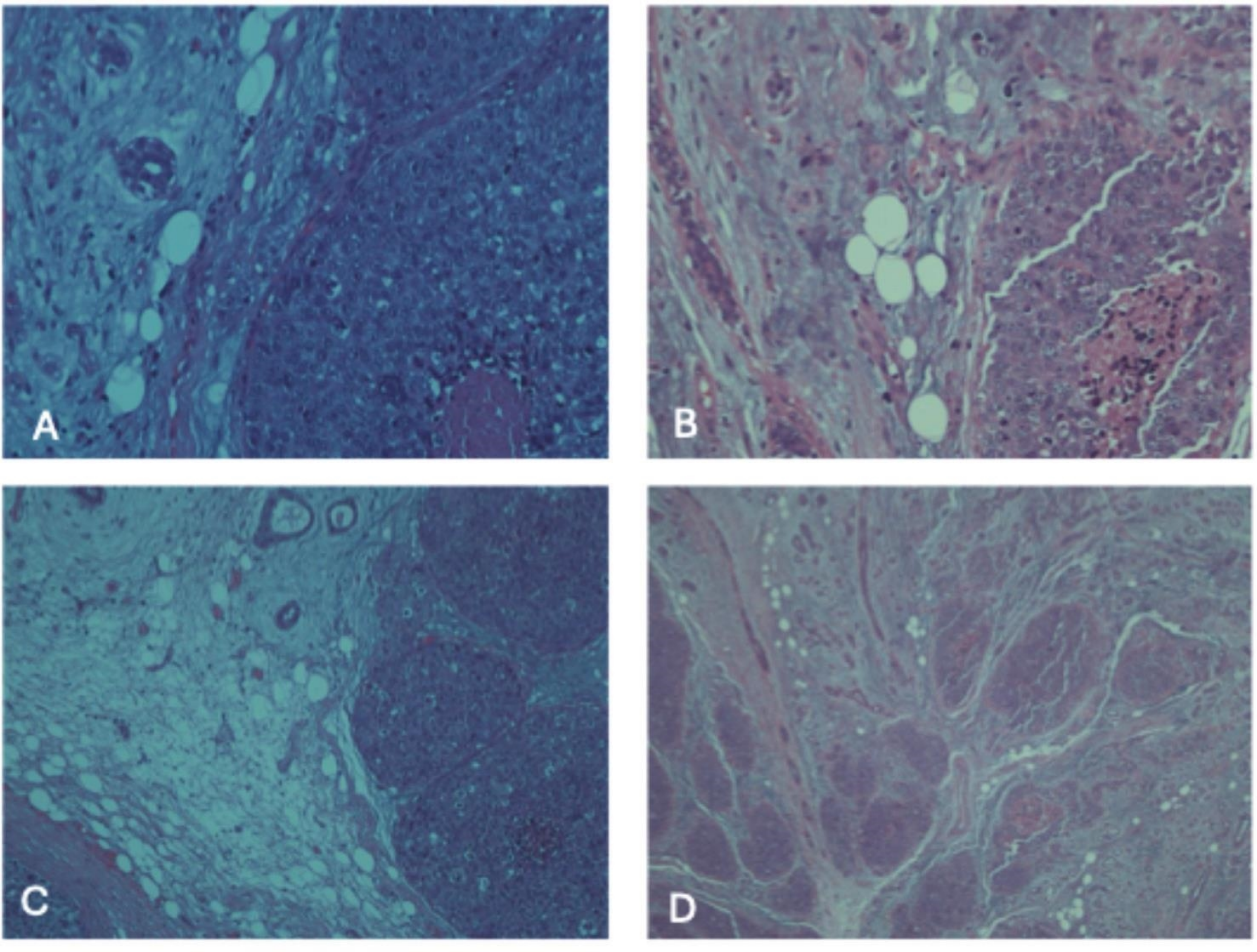
Further higher resolution microscopic images. (A) Hamartoma with DCIS. (B) Hamartoma with DCIS ×20 magnification. (C) Hamartoma left, DCIS right. (D) Hamartoma with DCIS ×4 magnification.

The separately resected retroareolar tissue shows benign breast glandular epithelium and lactiferous ducts; there was no evidence of in situ or invasive malignancy. Two sentinel lymph nodes were free of metastatic malignancy. The final staging was Tis N0 M0. There were no post‐operative side effects, and the aesthetic outcome was extremely satisfactory.

Unexpectedly, the patient developed a mass behind her left nipple–areolar complex in the subcutaneous plane between the skin and the implant capsule 6 months after her surgery (Figures 9 and 10). Biopsy demonstrated a triple‐negative grade 3 invasive ductal carcinoma and staging PETCT showed no evidence of distant metastases. She was treated with systemic anticancer therapy (KEYNOTE‐522 carbotaxol and EC chemotherapy with concurrent pembrolizumab) [16] as a neoadjuvant approach and underwent wide local excision of the nipple–areolar complex 6 months later showing a complete pathological response. This was followed by radiotherapy to her left chest wall and reconstruction (26 Gy in 5 fractions over 5 days), with a 5 mm bolus given the proximity of the recurrent lesion to the skin. She completed 1 year of pembrolizumab and continues to be in remission at 1 year following her surgery. Genetic testing was undertaken, and there was no evidence of a hereditary (germline) genetic cause for the patients’ breast cancer identified.

**Figure 9:**
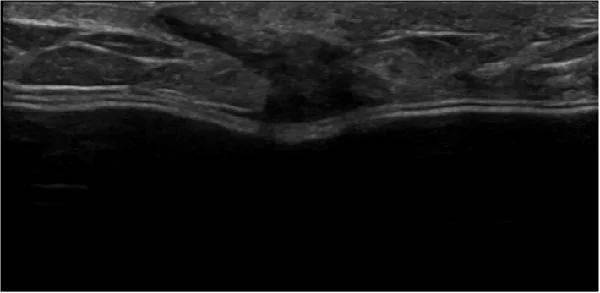
Ultrasound of breast reconstruction. Ultrasound 6 months post‐operatively in December 2024. This shows left sided mastectomy with implant reconstruction. Furthermore, in the central left breast there is a 2.3 cm hypoechoic lesion with moderate degree internal vascularity, which was seen to be contiguous with the nipple base anteriorly and extending over the anterior margin of the implant posteriorly. This was targeted for a core biopsy under ultrasound guidance as appearances were not typical of scarring given the internal vascularity.

**Figure 10:**
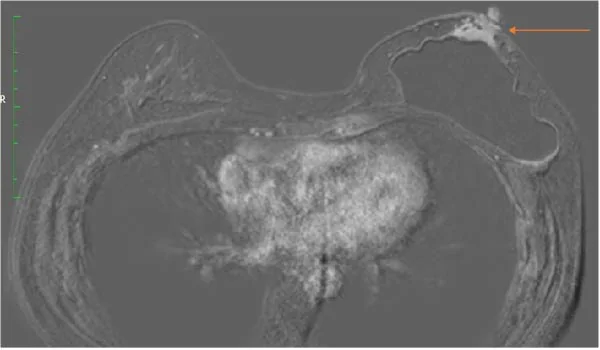
MRI breast post breast reconstruction. Post‐contrast subtracted sequence, post implant‐based reconstruction. This shows in the left breast (indicated by red arrow) deep to the nipple there is early and avid enhancement of a suspicious irregular lesion, measuring 2.8 cm. This lesion is confluent with the anterior margin of the implant and extends to the nipple base.

## 3. Discussion

Hamartomas result from haphazard growth of normal tissue and are an aggregation of cells from the local site [1], regarding the breast, this tends to contain varying amounts of fatty, fibrous and glandular elements [14]. They are usually asymptomatic and found incidentally on screening mammograms, or the patient may occasionally present to breast clinicians with palpable masses, which can cause significant anxiety for the patient. They are felt to be under‐diagnosed by pathologists, as the histopathological picture is not distinctive, especially with fine needle aspiration or core needle biopsy samples, as they may misrepresent the sample. Many can be reported as fibroadenomas, although hamartomas possess more glands, lobules, fat, and less stromal cellularity than a fibroadenoma. This may subsequently result in repeated biopsies or surgical excision of the lesion, which can be distressing for the patient. Hamartomas can have characteristic mammographic findings as thinly encapsulated masses with smooth borders containing fat density [7]. On ultrasonography, fat with echogenic fibrosis components is usually found; further diagnostics such as magnetic resonance imaging (MRI) also show characteristic features, such as a well‐defined hypointense rim, internal heterogenous enhancement and the presence of fat density; however, this modality is expensive and not readily available [7].

The pathogenesis of hamartomas is not fully clear; however, female sex hormones have been implicated in their development [7]. In a case series by Herbert et al. [17], in 24 hamartomas, the immunohistochemical analysis revealed there was oestrogen and PR positivity in the epithelial and stromal cells, possibly indicating some proliferative activity. Work by Panagopoulos et al provided the first‐time genetic characterisation of a breast myoid hamartoma, demonstrating rearrangement of the HMGA2 (12q14.3) locus, the tumour cells had t(5;12)(p13;q14) as the only cytogenetic abnormality. The translocation led to rearrangement of the HMGA2 gene, fusing it with a sequence from chromosome band 5p13, a finding that mirrors genetic alterations observed in other benign and more frequent connective tissue tumours [10]. These findings suggest that myoid hamartomas arise from mesenchymal stem cells capable of smooth muscle differentiation that acquire genetic alterations, supporting their classification as true clonal neoplasms rather than developmental malformations [9].

Subsequently, to this Sta. Ines et al. [9] have published the first known in situ and invasive carcinoma found in a myoid hamartoma. The lesion exhibited the same HMGA2 genetic aberration described earlier by Panagopoulos et al. [10] which supports the hypothesis of smooth muscle cell differentiation of the stromal cells; this strengthens the association between HMGA2 rearrangement, HMGA2 protein overexpression, and the development of breast myoid hamartoma [9]. In this instance, the authors have highlighted that HMGA2 may become a valuable diagnostic biomarker, particularly in difficult core biopsies where distinguishing myoid hamartomas from other spindle cell or mesenchymal lesions can be difficult [9].

Hamartomas are usually benign, but malignant transformation may occur in some cases. We know that mutations in the PTEN gene result in its loss of function, resulting in PTEN hamartoma tumour syndrome, where multiple hamartomas can develop throughout the body. A well‐documented syndrome is Cowden’s, where there is an increased risk of breast, thyroid, and endometrial cancer [1].

As previously highlighted, the incidence of breast hamartoma among benign breast tumours is usually reported around 4%. In a large case series, the average age of patients ranged between 13 and 89 years with a mean of 37.8 years; further to this, the mean patient age of those to have malignancy within a hamartoma was 58.2 [7]. Malignancies associated with breast hamartomas are rarely observed, and only 16 case studies have reported such events [14, 15, 18]. We are unable to accurately calculate the incidence, as a majority of the literature reports are individual case reports. Some larger case series of benign hamartomas have reported breast cancer findings but these cannot accurately represent the overall incidence. In a case series by Sevim et al. [18], out of 27 hamartomas, only 1 case was found to be invasive ductal carcinoma, ductal epithelial hyperplasia was identified in 22.2% of cases; similarly, Wahner‐Rodler et al. [19] reported the incidence rate as 26% in their case series of 35 patients. These findings question whether should be undertaking excisional surgery on these patients, as clearly cellular level changes are occurring.

We currently have no specific national or international guidelines from the pathology, radiology or breast surgery communities focusing on hamartoma management. The Royal College of Radiologists, England, does state that if a presumed lipoma or hamartoma is detected, if deemed P2 and imaging is typical, no biopsy is required. If there is any doubt about the nature of the lesion, or if there is a discrepancy between imaging and clinical features, then needle biopsy should be performed [20]. The American College of Radiology recommends that if the mammogram shows a definite benign mass (e.g., hamartoma), ultrasound is not necessary as long as the benign mass identified on mammography is a definitive correlate of the clinical finding [21]. The American Society of Breast Surgery provides guidance on when to start MRI screening in women with the germline mutation PTEN hamartoma tumour syndrome‐PT53 as it is known to predispose to a high risk of breast cancer [22]. However, there is no further management pathway recommendation for hamartoma in their benign breast disease guidelines [21]. Neither the Association of Breast Surgery nor the European guidelines on B3 lesions comment on hamartomas specifically [23].

Although breast hamartomas are rare and mainly benign lesions, the under‐recognition and hence underdiagnosis along with the suggestion of possible malignant transformation indicate the need for further investigation. Until the association between malignancy and hamartoma is further established, it is vital that a representative histopathological sample should be undertaken. Thus, the true incidence may be higher than the literature indicates. Furthermore, we highlight the importance of molecular characterisation of these lesions. Further genetic profiling may determine whether certain hamartomas share molecular features with other benign mesenchymal tumours, providing a greater insight into their pathogenesis and biological behaviour.

Currently, there are only generic guidelines and recommendations from the literature on how to manage hamartomas, and it seems to be conflicting. There is no consensus whether imaging alone is suitable for diagnosis, in combination with histology or whether histological evidence is necessary [5]. To the inexperienced radiological eye, abnormalities within these complex and rare lesions may be overlooked, and the opportunity to identify a malignant process is therefore missed [24].

Larger‐sized lesions should warrant additional imaging such as mammograms even in patients under the age of 40. In our patient, mammograms clearly identified malignant calcifications. Other imaging modalities such as MRI can also be considered to better characterise large lesions, but the literature on this is limited. Surgeons should discuss the excision of larger hamartomas despite benign imaging as biopsies may not be representative. However, this can present considerable challenges to breast‐conserving surgery and the potential cosmetic impact can be off‐putting to some patients. This was one of the reasons why our patient avoided surgery on her large breast lump for over 10 years.

Our patient’s development of a new invasive breast cancer under her nipple was highly unexpected. She had a well‐demarcated hamartoma within her mastectomy specimen, and furthermore, the DCIS component was entirely contained within the hamartoma and therefore fully excised. The DCIS component was located towards the posterior aspect of the hamartoma and was therefore distant from her skin and left nipple, with anterior margins in excess of 10 mm. Additional tissue sampling behind the left nipple at the time of mastectomy revealed normal ducts. On the basis of her having a mastectomy containing ER‐negative DCIS only, no adjuvant therapy was felt necessary. The presence of microinvasion in the preoperative biopsies was noted but not felt to justify adjuvant systemic therapy.

Due to the short time frame between mastectomy and the development of a new mass behind her left nipple, it is felt that her new invasive cancer developed in the residual breast tissue not related to the hamartoma. At the time of her initial presentation and mastectomy, this invasive disease near the nipple was occult to imaging and was not identified in the anterior margins of the mastectomy. Fortunately, she responded extremely well to neoadjuvant anticancer therapy, and following subsequent wide local excision and adjuvant radiotherapy, she remains in remission.

A central database capturing the incidence of hamartomas and subsequent pathological findings, including malignancy, will give a better overview of the incidence rate. It has not yet become clear on how and when we should follow these patients up, especially if surgical intervention is not performed. Although rare, it is necessary to consider the possibility of a malignant tumour inside a hamartoma, and for that reason, we may need to consider surgical excision as a recommendation.

## Funding

No funding or grant support has been provided.

## Conflicts of Interest

The authors declare no conflicts of interest.

## Data Availability

Research data are not shared.
